# Supplementary material for: Poor lie detection related to an under-reliance on statistical cues and overreliance on own behaviour
Source: Commun Psychol. 2024 Mar 14;2:21. doi: 10.1038/s44271-024-00068-7 (PMC11332128; doi:10.1038/s44271-024-00068-7)
Supplement: Supplementary file 1 — Supplementary Information [file 44271_2024_68_MOESM1_ESM.docx]

# Supplementary Information for “Poor lie detection related to an under-reliance on statistical cues and overreliance on own behaviour”

## **Supplementary Figure 1: Experiment 1 violin plots**


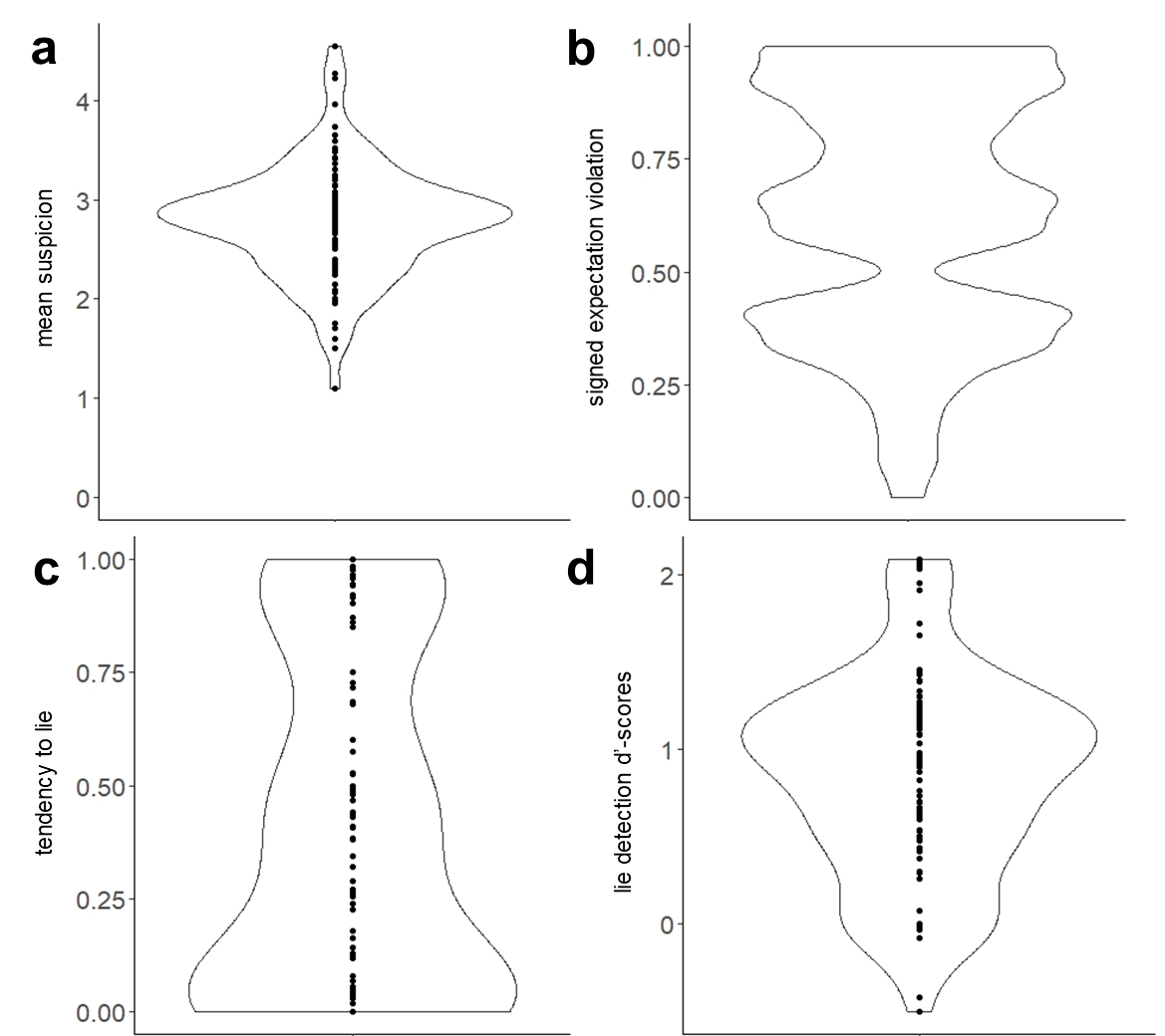


**a.** Distribution of participants’ mean suspicion (i.e., the raw reverse-coded honesty ratings averaged across all trials; N_participants_ = 102). **b.** Distribution of signed expectation violations across all trials and participants (N_trials_ = 9180). **c.** Distribution of proportion of trials on which participants lied (N = 102). **d.** Distribution of participants’ d’-scores indicating their ability to detect lies (N = 102). Dots represent individual participants.

## **Supplementary Figure 2: Experiment 2 violin plots**

**
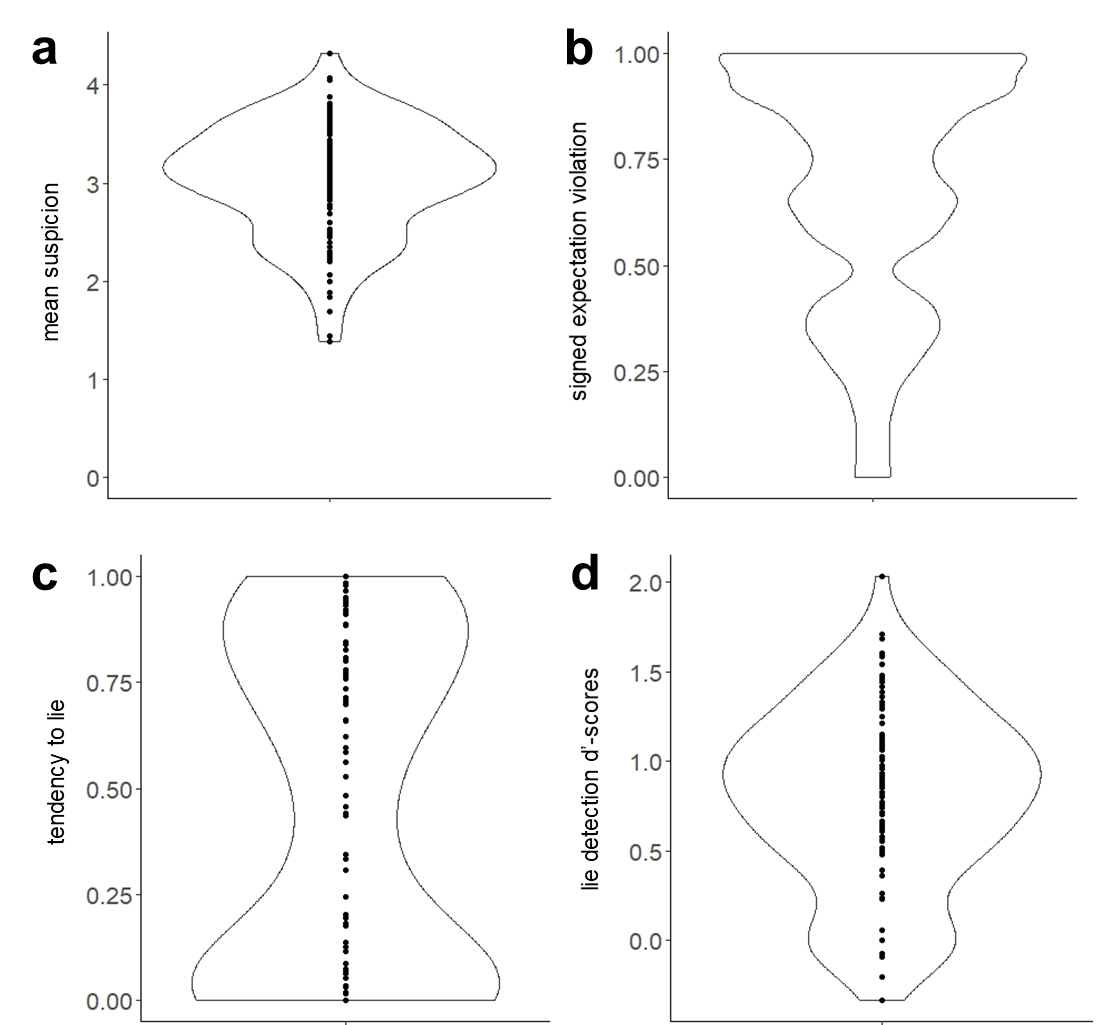
**

**a.** Distribution of participants’ mean suspicion (i.e., the raw reverse-coded honesty ratings averaged across all trials; N_participants_ = 108). **b.** Distribution of signed expectation violations levels across all trials and participants (N_trials_ = 9720). **c.** Distribution of proportion of trials on which participants lied (N = 108). **d.** Distribution of participants’ d’-scores indicating their ability to detect lies (N = 108). Dots represent individual participants.

## **Supplementary Figure 3: Experiment 3 violin plots**


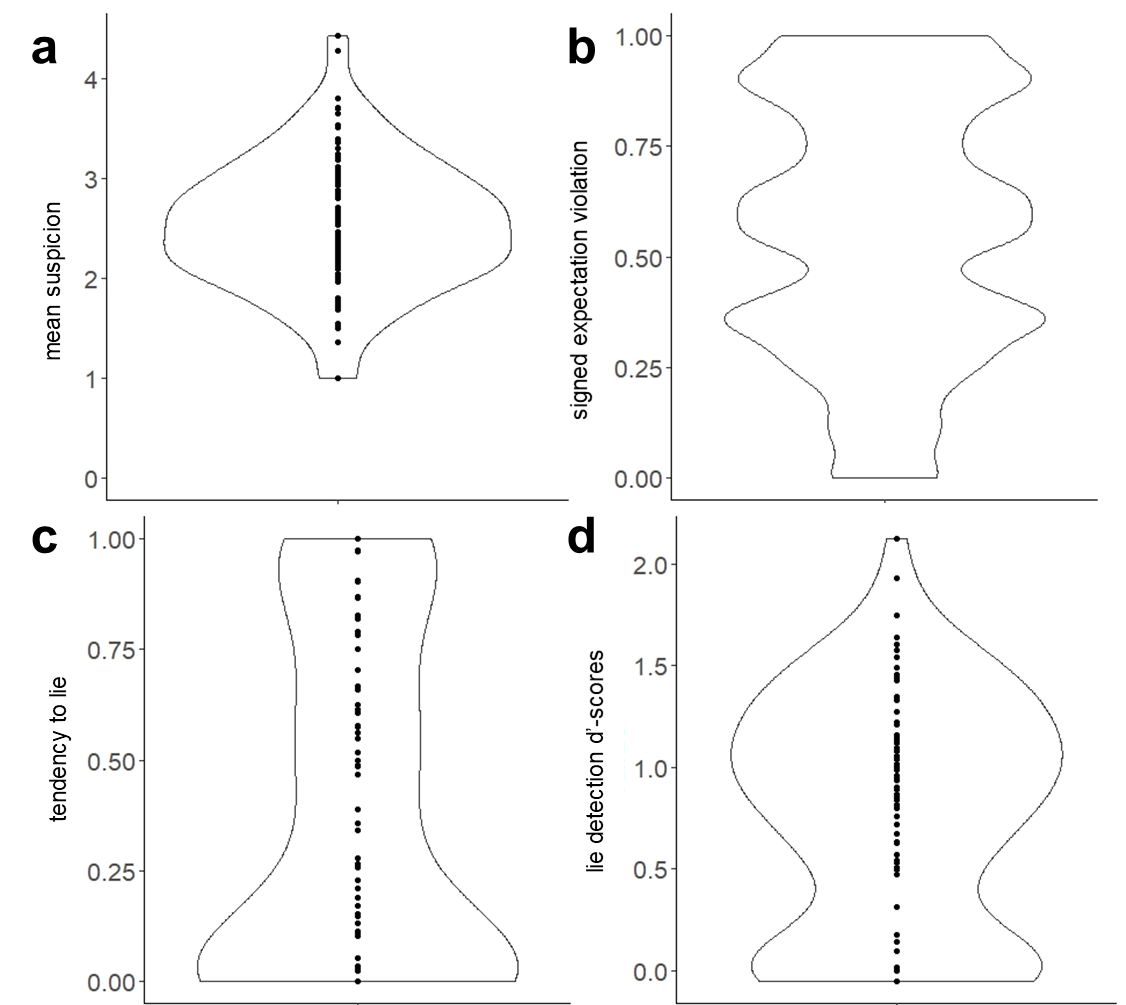


**a.** Distribution of participants’ mean suspicion (i.e., the raw reverse-coded honesty ratings averaged across all trials; N_participants_ = 100). **b.** Distribution of signed expectation violations levels across all trials and participants (N_trials_ = 6000). **c.** Distribution of proportion of trials on which participants lied (N = 100). **d.** Distribution of participants’ d’-scores indicating their ability to detect lies (N = 100). Dots represent individual participants.

**Supplementary Notes 1: Post-task questions**

Experiment 1

All participants were asked:

To what extent did you feel you understood the game instructions? (7-point Likert scale “Not at all” – “Completely understood”)

- Participants felt they understood the task instructions well (M=5.83, median=6, SD=1.45).

How many different participants do you think you ended up playing with?

- Seven participants thought they did not see responses from past participants, but computer-generated responses. See full histogram below.


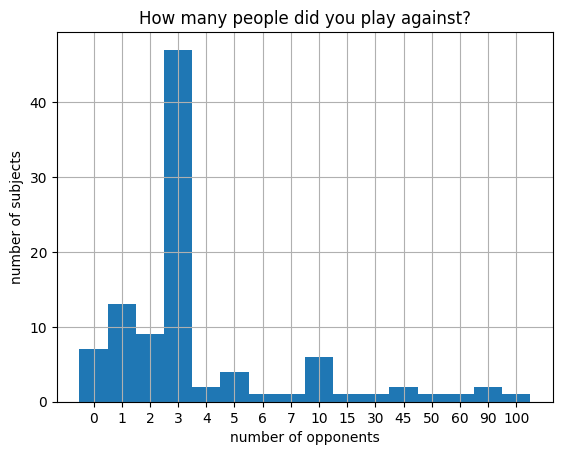


What do you think was the purpose of this study?

- 56 participants thought the purpose of the study related to honesty, 30 participants thought it was a purpose unrelated to honesty and 13 participants said they didn’t know.

What was your goal while playing the game?

- 37 participants said their goal was to report honestly throughout the task, 10 participants said they remained mostly honest and only lied in certain cases, 57 participants said their goal was to win as much as possible or at least to avoid losing, 18 participants said they tried to analyse others’ responses to determine a good strategy, 4 participants said they were trying to figure out the purpose of the experiment as a whole. Some of these participants mentioned multiple goals, hence summing these counts gives a higher total than the number of participants in Experiment 1 who completed all post-task questions (N=99).

What do you think the other players' goals were in playing the game?

- 12 participants thought the other players’ goal was to report honestly throughout the task, 22 participants thought the other players remained mostly honest and only lied in certain cases, 74 participants thought the other players’ goal was to win as much as possible or at least to avoid losing, 6 participants thought the other players tried to analyse their responses to determine a good strategy, 4 participants thought the other players’ goal was to figure out the purpose of the experiment as a whole.

Would you play this game again?

- Out of the 99 participants who completed all post-task questions, 79 said they would play the cards game task again and 20 said they would not.

How honest were you in playing the game? (7-point Likert scale “Not honest at all” – “Completely honest”)

- Participants said on average they were mostly honest (M=4.61, median=5, SD=2.05).

How good do you think you are at detecting deceptions? (7-point Likert scale “Not good at all” – “Very good”)

- Participants thought on average they were somewhat good at detecting deceptions (M=4.36, median=4, SD=1.31).

Experiment 2

All participants were asked:

To what extent did you feel you understood the game instructions? (7-point Likert scale “Not at all” – “Completely understood”)

- Participants felt they understood the task instructions well (M=6.07, median=6, SD=1.18).

How many different participants do you think you ended up playing with?

- Seventeen participants thought they did not see responses from past participants, but computer-generated responses. See full histogram below.


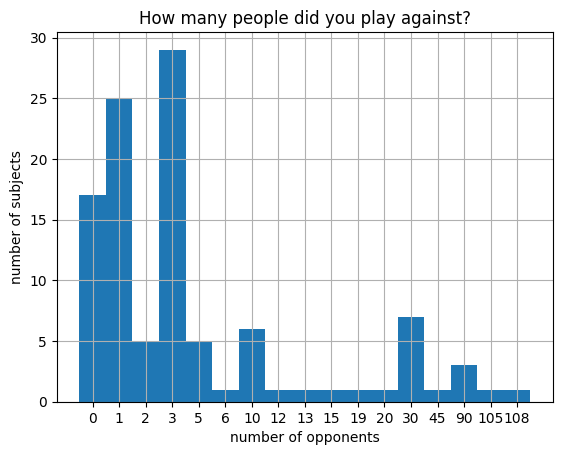


What do you think was the purpose of this study?

- 77 participants thought the purpose of the study related to honesty, 21 participants thought it was a purpose unrelated to honesty and eight participants said they didn’t know.

What was your goal while playing the game?

- 29 participants said their goal was to report honestly throughout the task, 8 participants said they remained mostly honest and only lied in certain cases, 75 participants said their goal was to win as much as possible or at least to avoid losing, 11 participants said they tried to analyse others’ responses to determine a good strategy, 3 participants said were trying to figure out the purpose of the experiment as a whole. Some of these participants mentioned multiple goals, hence summing these counts gives a higher total than the number of participants in Experiment 2.

What do you think the other players' goals were in playing the game?

- 7 participants thought the other players’ goal was to report honestly throughout the task, 18 participants thought the other players remained mostly honest and only lied in certain cases, 93 participants thought the other players’ goal was to win as much as possible or at least to avoid losing, 7 participants thought the other players (also) tried to analyse their responses to determine a good strategy.

Would you play this game again?

- 78 participants said they would play the cards task again and 28 said they would not.

How honest were you in playing the game? (7-point Likert scale “Not honest at all” – “Completely honest”)

- Participants said on average they were somewhat honest themselves (M=4.12, median=4, SD=2.25).

How good do you think you are at detecting deceptions? (7-point Likert scale “Not good at all” – “Very good”)

- Participants thought on average they were somewhat good at detecting deceptions (M=4.27, median=4, SD=1.31).

Experiment 3

All participants were asked:

To what extent did you feel you understood the game instructions? (7-point Likert scale “Not at all” – “Completely understood”)

- Participants felt they understood the task instructions well (M=5.96, median=6, SD=1.23).

How many different participants do you think you ended up playing with?

- Twenty-one participants thought they did not see responses from past participants, but computer-generated responses. See full histogram below.


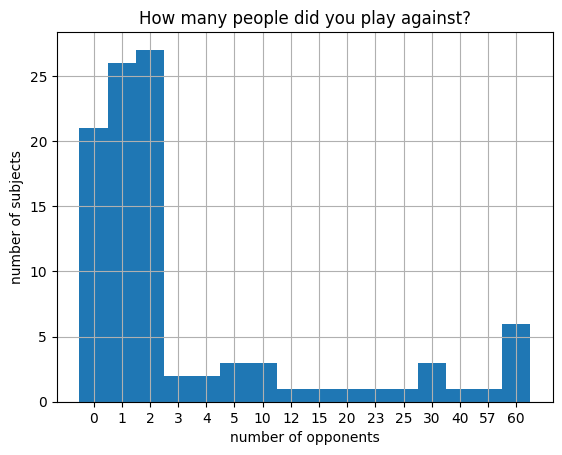


What do you think was the purpose of this study?

- 69 participants thought the purpose of the study related to honesty, 20 participants thought it was a purpose unrelated to honesty and 11 participants said they didn’t know.

What was your goal while playing the game?

- 32 participants said their goal was to report honestly throughout the task, 9 participants said they remained mostly honest and only lied in certain cases, 61 participants said their goal was to win as much as possible or at least to avoid losing, 11 participants said they tried to analyse others’ responses to determine a good strategy, 2 participants said were trying to figure out the purpose of the experiment as a whole.

What do you think the other players' goals were in playing the game?

- 14 participants thought the other players’ goal was to report honestly throughout the task, 9 participants thought the other players remained mostly honest and only lied in certain cases, 70 participants thought the other players’ goal was to win as much as possible or at least to avoid losing, 3 participants thought the other players (also) tried to analyse their responses to determine a good strategy.

Would you play this game again?

- All 100 participants completed all post-task questions. Out of these, 65 said they would play the cards game task again and 35 said they would not.

How honest were you in playing the game? (7-point Likert scale “Not honest at all” – “Completely honest”)

- Participants said on average they were somewhat honest themselves (M=4.65, median=5, SD=2.29).

How good do you think you are at detecting deceptions? (7-point Likert scale “Not good at all” – “Very good”)

- Participants thought on average they were somewhat good at detecting deceptions (M=4.11, median=4, SD=1.39).

**Supplementary Tables 1: Correlations between suspicion cues**

a. Experiment 1

| Pearson’s r | Unsigned expectation violation | Signed expectation violation | Lying oneself |
| --- | --- | --- | --- |
| Unsigned expectation violation | 1 |  |  |
| Signed expectation violation | 0.558** | 1 |  |
| Lying oneself | 0.08 | 0.047 | 1 |
| Losing | 0.418 | 0.486* | -0.148 |

b. Experiment 2

| Pearson’s r | Unsigned expectation violation | Signed expectation violation | Lying oneself |
| --- | --- | --- | --- |
| Unsigned expectation violation | 1 |  |  |
| Signed expectation violation | 0.638** | 1 |  |
| Lying oneself | 0.1 | 0.084 | 1 |
| Losing | 0.366 | 0.43 | -0.155 |

c. Experiment 3

| Pearson’s r | Unsigned expectation violation | Signed expectation violation | Lying oneself |
| --- | --- | --- | --- |
| Unsigned expectation violation | 1 |  |  |
| Signed expectation violation | 0.556* | 1 |  |
| Lying oneself | 0.077 | 0.025 | 1 |
| Losing | 0.425 | 0.505* | -0.138 |

To check for the relationship between predictors, we performed Pearson’s correlation tests for every combination of the four cues for each participant. We then used one-sample t-tests to examine if the obtained correlation coefficients were significantly different from zero. As observed in these tables**,** correlations between predictors ranged between small to medium size, which allowed model fitting.

* p < .05, ** p < .001

**Supplementary Tables 2: Leave-one-out cross validation**

a. Participants:

| Cue | Cross-validated mean weighted β [mean 95%-CI lower bound; mean 95%-CI upper bound] | | |
| --- | --- | --- | --- |
|  | Experiment 1 | Experiment 2 | Experiment 3 |
| Lying oneself | 0.043 [0.033; 0.054] | 0.09 [0.068; 0.112] | 0.079 [0.06; 0.099] |
| Signed expectation violation | 0.354 [0.267; 0.44] | 0.345 [0.26; 0.429] | 0.31 [0.234; 0.385] |
| Unsigned expectation violation | 0.197 [0.149; 0.245] | 0.186 [0.141; 0.232] | 0.194 [0.146; 0.241] |
| Losing | 0.053 [0.04; 0.066] | 0.05 [0.038; 0.062] | 0.087 [0.065; 0.108] |

b Accurate lie detector:

| Cue | Cross-validated mean weighted β [mean 95%-CI lower bound; mean 95%-CI upper bound] | | |
| --- | --- | --- | --- |
|  | Experiment 1 | Experiment 2 | Experiment 3 |
| Lying oneself | 0 [0; 0] | 0 [0; 0] | 0.001 [0.001; 0.001] |
| Signed expectation violation | 0.295 [0.225; 0.365] | 0.329 [0.25; 0.407] | 0.305 [0.256; 0.354] |
| Unsigned expectation violation | 0.313 [0.238; 387] | 0.351 [0.267; 0.435] | 0.336 [0.282; 0.39] |
| Losing | 0 [0; 0] | 0 [0; 0] | 0.025 [0.019; 0.03] |

We performed leave-one-out cross-validation with the same Bayesian averaging procedure on the four cues to predict participants’ and the accurate lie detector’s suspicion ratings. As in the main results, we deemed a cue significant if its mean cross-validated 95% confidence interval did not include zero. The results confirm the main analyses in all experiments.

**Supplementary Tables 3: Suspicion modelling results after excluding participants who did not believe they saw past participants’ responses**

| Cue | Mean weighted β [95%-CI lower bound; 95%-CI upper bound] | | |
| --- | --- | --- | --- |
|  | Experiment 1 | Experiment 2 | Experiment 3 |
| Lying oneself | 0.046 [0.058; 0.035] | 0.092 [0.069; 0.114] | 0.066 [0.05; 0.082] |
| Signed expectation violation | 0.35 [0.264; 0.436] | 0.337 [0.254; 0.419] | 0.32 [0.241; 0.398] |
| Unsigned expectation violation | 0.196 [0.148; 0.244] | 0.191 [0.144; 0.238] | 0.185 [0.14; 0.23] |
| Losing | 0.052 [0.039; 0.065] | 0.052 [0.39; 0.065] | 0.085 [0.064; 0.106] |

We replicated all model fit analyses for each experiment after excluding participants who answered zero to the post-task question “How many different participants do you think you ended up playing with?” (Experiment 1: N = 7, Experiment 2: N = 17, Experiment 3: N = 21). As none of the 95%-CIs include zero, the conclusion that participants use all four cues when judging others’ honesty, including their own lying behaviour, remains unchanged.

**Supplementary Tables 4: Proportion for whom each model predictor is significantly positive**

| Cue | Proportion of participants where cue estimate was significant | | |
| --- | --- | --- | --- |
|  | Experiment 1 | Experiment 2 | Experiment 3 |
| Lying oneself | 62.8% | 71.6% | 67.1% |
| Signed expectation violation | 89.2% | 91.7% | 91% |
| Unsigned expectation violation | 83.3% | 86.1% | 81% |
| Losing | 65.2% | 60.8% | 67.8% |

We report for how many participants each fitted cue was significantly positive (i.e., 95%-CIs above zero) after applying Bayesian model averaging on each participant’s data.

**Supplementary Notes 2: Alternative lie detection metrics**

Honesty judgement accuracy

We also compared the overall judgement accuracy of humans with that of accurate lie detector model. Again, we dichotomised participants’ suspicion ratings to 1 if they thought the other person was (completely/somewhat) dishonest, or 0 if they thought the other person was (completely/somewhat) honest. Then we used the accurate lie detector’s overall model to predict the honesty in each participant’s trials set. We computed both humans and accurate lie detectors’ honesty judgement accuracy as

tp + tn / N_trials_,

where *tp* reflects true positives (i.e., the participant or accurate lie detector correctly judged a trial as dishonest), *tn* reflects true negatives (i.e., the participant or accurate lie detector correctly judged a trial as honest) and N_trials_ denotes the total number of trials in each participant’s trials set (i.e., 90 in Experiment 1 and 2, and 60 in Experiment 3). We found that participants’ average accuracy is 0.725 (SD = 0.095) in Experiment 1, 0.631 (SD = 0.085) in Experiment 2 and 0.748 (SD = 0.08) in Experiment 3. The accurate lie detector’s average accuracy is 0.806 (SD = 0.05) in Experiment 1, 0.777 (SD = 0.042) in Experiment 2 and 0.845 (SD = 0.036) in Experiment 3. We then compared the accurate detector’s accuracy with that of participants and find that the accurate detector is more accurate at discerning honesty in Experiment 1 (Wilcoxon signed rank test = 4596, p < .001), Experiment 2 (Wilcoxon signed rank test = 5535.5, p < .001) and Experiment 3 (Wilcoxon signed rank test = 4365.5, p < .001).

Next, as for d’-scores, we ran linear regressions predicting participants’ overall accuracy from their demographics, psychometric scores and their betas for each of the four suspicion cues. We found that accuracy is related to higher betas for signed expectation violation in Experiment 1 (standardized β = 0.303, t(89) = 2.92, p = .004), Experiment 2 (standardized β = 0.715, t(98) = 8.64, p < .001) and Experiment 3 (standardized β = 0.212, t(90) = 2.07, p = .041) and unsigned expectation violation in Experiment 1 (standardized β = 0.553, t(89) = 6.18, p < .001), Experiment 2 (standardized β = 0.525, t(98) = 8.17, p < .001) and Experiment 3 (standardized β = 0.371, t(90) = 3.89, p < .001), but not betas for when participants lied themselves in Experiment 1 (standardized β = 0.086. t(89) = 1, p = .319), Experiment 2 (standardized β = 0.002. t(98) = 0.03, p = .973) and Experiment 3 (standardized β = -0.044. t(90) = -0.46, p = .649), None of the other predictors significantly related to accuracy in all three experiments. Thus, in line with our linear regression results predicting d’-scores, we consistently find in all experiments that participants who rely more on statistical cues are better at discerning others’ honesty and that participants’ betas for their own lying are not related to discernment (whether measured as accuracy or d’-scores).

Lie detection bias predictors

To examine participants’ overall tendency to “detect” lies, we also compute SDT’s beta criterion according to the following formula:

beta criterion = exp(-z(H) * z(H) / 2 + z(FA) * z(FA) / 2).

The beta criterion reflects a participant’s bias to “detect” a lie (i.e., when they rated the other person’s report as (somewhat/completely) dishonest). A participant who is overly suspicious will “detect” more lies, which results in higher hits (H) and higher false alarms (FA). As H and FA both increase, beta will approach zero. Vice versa, someone who tends to believe others will have lower hit and false alarm rates and therefore a beta criterion of around 1 or higher.

We then ran a linear regression predicting participants’ beta criterions from their individual beta coefficients in relation to suspicion, demographics (age, gender, education level), psychological traits (cognitive reflection in all experiments; Experiment 1 also includes autism, empathy and paranoia) and how often they lied themselves. We found that higher betas relating suspicion to unsigned expectation violation predicted higher betas in Experiment 2 (standardised β = 0.316, t(98) = 3.39, p = .001) and Experiment 3 (standardised β = 0.363, t(90) = 3.71, p < .001), with this effect at trending level in Experiment 1 (standardised β = 0.182, t(89) = 1.74, p = .085). None of the other predictors significantly related to beta criterions across the three experiments.

**Supplementary Notes 3: Brunswik’s lens model analysis**

Brunswik’s lens model (Hammond & Stewart, 2001) provides an alternative framework to analyse what information sources humans versus an accurate lie detector use in judging honesty. We follow the adapted lens model for human judgement research as described by Cooksey (1996), by examining the correlations between cues and an observer’s judgments (i.e., ‘cue utilization validities), and compare these to the correlations between cues and an “ecological criterion” (i.e., ‘ecological validities’). We thus compute the Pearson’s correlation (r) between each of our four hypothesised information cues (one’s own lying behaviour (X_1_), signed expectation violation (X_2_), unsigned expectation violation (X_3_), losing (X_4_), the accurate lie detector’s suspicion, and human suspicion ratings in each experiment. Here, the *ecological criterion* (Y_e_; Cooksey, 1996) represents the accurate lie detector’s ground truth of whether the other person actually lied on a trial, and *human judgement* (Y_s_) represents participants’ suspicion ratings. See figures below.

Experiment 1


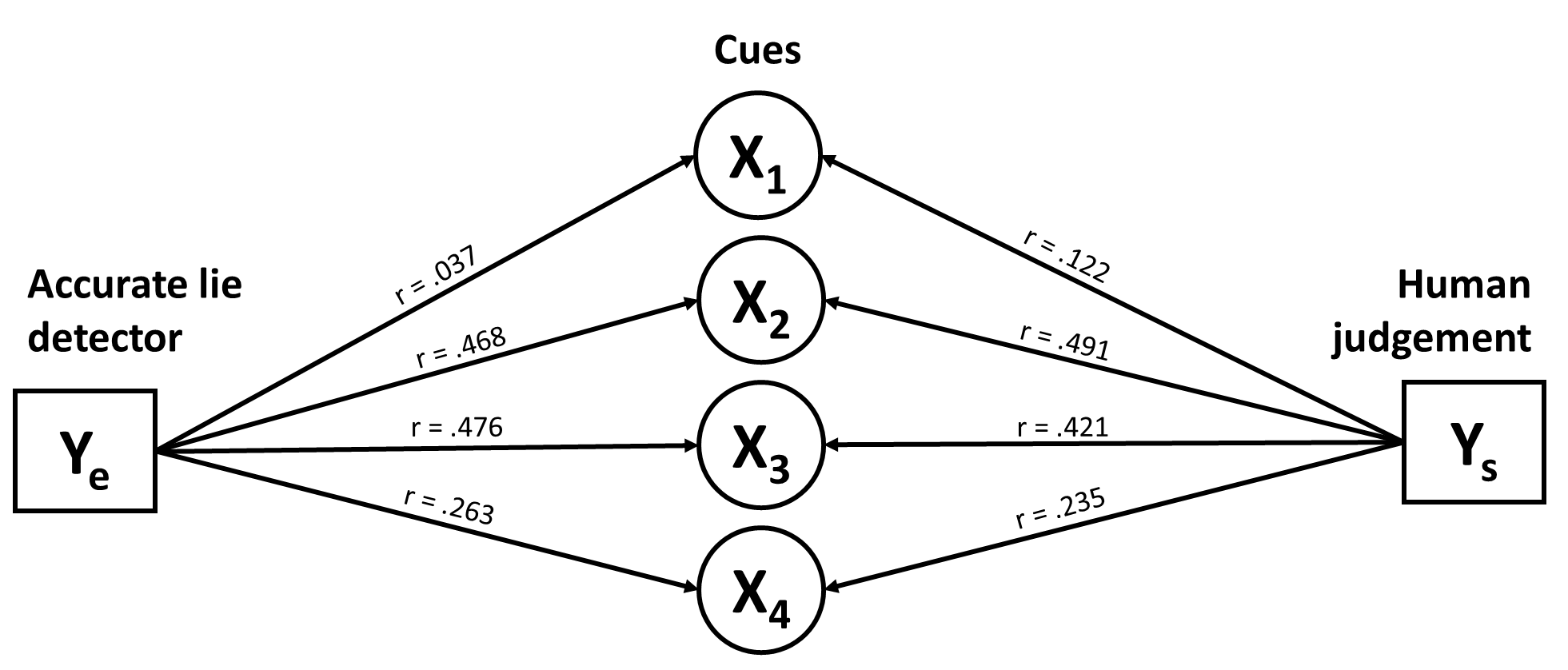


Experiment 2


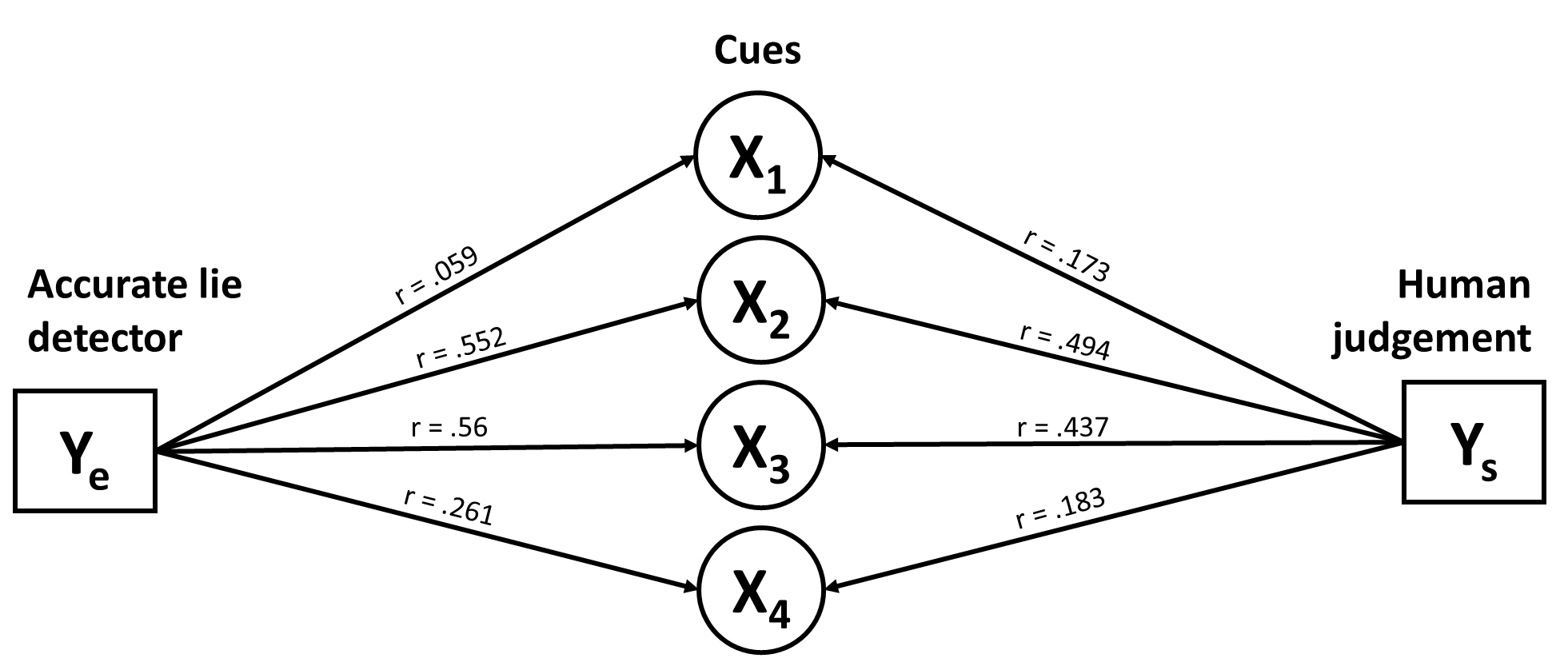


Experiment 3


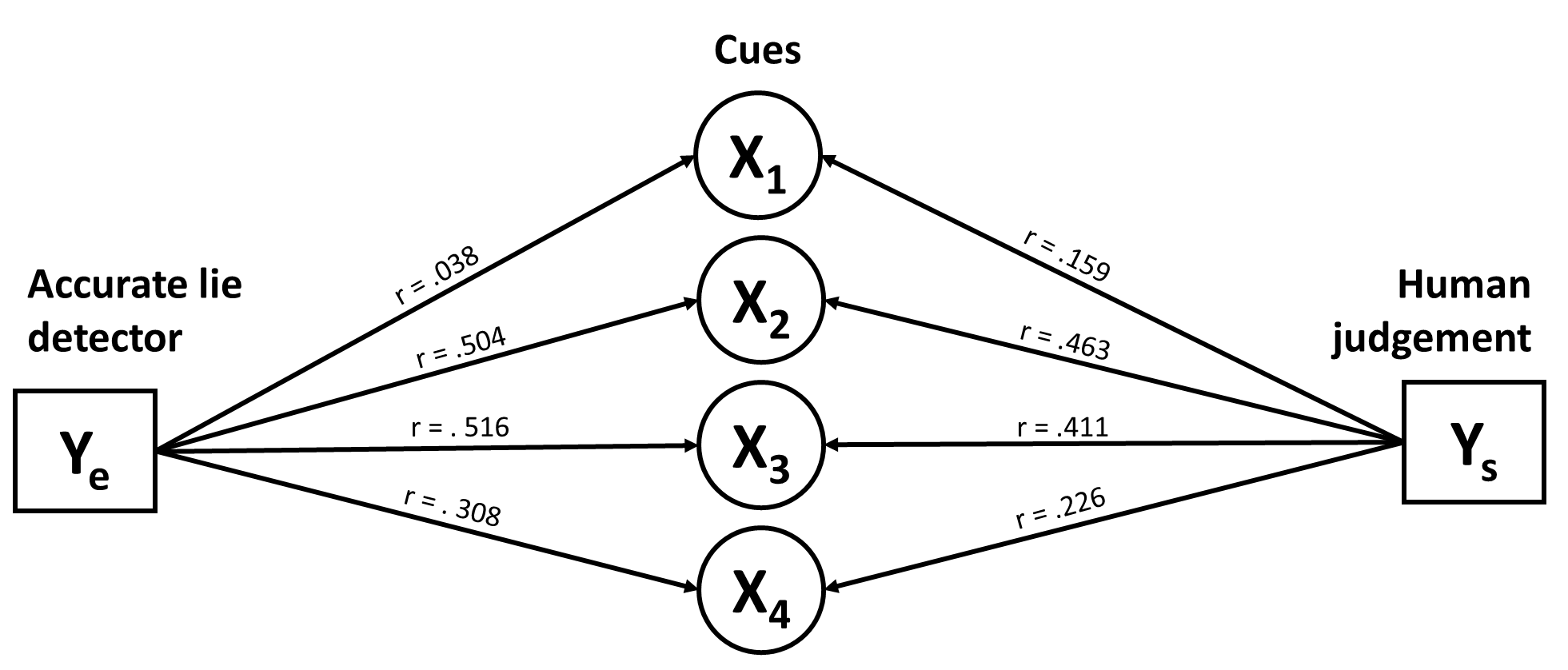


The results consistently show across the three experiments that information on whether participants themselves lied has a near-zero ecological validity (r_(Ye, X1)_ < .06), but much higher cue utilization validity (r_(Ys, X1)_ > .12). The ecological validities of the statistical cues (X_2_,_3_) are also higher compared to the cue utilization validities in nearly all cases across the three experiments. These findings are in line with the main Results.

**Supplementary Tables 5: Controlling for other’s reported colour in the Bayesian averaging model**

| Cue | Weighted β [mean 95%-CI lower bound; mean 95%-CI upper bound] | | |
| --- | --- | --- | --- |
|  | Experiment 1 | Experiment 2 | Experiment 3 |
| Lying oneself | 0.056 [0.049; 0.064] | 0.105 [0.093; 0.118] | 0.093 [0.081; 0.104] |
| Signed expectation violation | 0.254 [0.223; 0.286] | 0.226 [0.199; 0.254] | 0.226 [0.199; 0.254] |
| Unsigned expectation violation | 0.215 [0.189; 0.242] | 0.212 [0.186; 0.238] | 0.21 [0.184; 0.235] |
| Other person’s reported card colour | 0.1 [0.088; 0.112] | 0.114 [0.1; 0.128] | 0.088 [0.077; 0.098] |
| Losing | 0.057 [0.05; 0.064] | 0.058 [0.051; 0.065] | 0.091 [0.08; 0.102] |

Signed expectation violation is the product of the unsigned expectation violation and the colour reported by the other participant (+1 if blue and -1 if red). As such, the former can be considered an interaction effect, with the latter two considered main effects. In the main text, the colour reported by the other participant is not included as a factor on its own (but only when multiplied with the unsigned expectation violation). To test if the results are different when including this variable, we ran the Bayesian model averaging procedure on participants’ suspicion in each experiment again with this cue added. Doing so does not change the results.

**Supplementary References**

Cooksey, R.W. (1996) The Methodology of Social Judgement Theory, Thinking & Reasoning, 2, 141-174, https://doi.org/10.1080/135467896394483

Hammond, K.R., & R. Stewart, T. (2001). Essential Brunswik. Oxford University Press USA.
